# Effect of Formant Frequency Spacing on Perceived Gender in Pre-Pubertal Children's Voices

**DOI:** 10.1371/journal.pone.0081022

**Published:** 2013-12-03

**Authors:** Valentina Cartei, David Reby

**Affiliations:** School of Psychology, Sussex University, Brighton, United Kingdom; The University of Chicago, United States of America

## Abstract

**Background:**

It is usually possible to identify the sex of a pre-pubertal child from their voice, despite the absence of sex differences in fundamental frequency at these ages. While it has been suggested that the overall spacing between formants (formant frequency spacing - ΔF) is a key component of the expression and perception of sex in children's voices, the effect of its continuous variation on sex and gender attribution has not yet been investigated.

**Methodology/Principal findings:**

In the present study we manipulated voice ΔF of eight year olds (two boys and two girls) along continua covering the observed variation of this parameter in pre-pubertal voices, and assessed the effect of this variation on adult ratings of speakers' sex and gender in two separate experiments. In the first experiment (sex identification) adults were asked to categorise the voice as either male or female. The resulting identification function exhibited a gradual slope from male to female voice categories. In the second experiment (gender rating), adults rated the voices on a continuum from “masculine boy” to “feminine girl”, gradually decreasing their masculinity ratings as ΔF increased.

**Conclusions/Significance:**

These results indicate that the role of ΔF in voice gender perception, which has been reported in adult voices, extends to pre-pubertal children's voices: variation in ΔF not only affects the perceived sex, but also the perceived masculinity or femininity of the speaker. We discuss the implications of these observations for the expression and perception of gender in children's voices given the absence of anatomical dimorphism in overall vocal tract length before puberty.

## Introduction

Adults can discriminate the sex of adult [1] and of children [2], [3] speakers by listening to their voice only. Sex identification in adult voices is substantially determined by acoustic differences in fundamental frequency (F0) and in the overall pattern of formant frequencies (ΔF, or formant spacing), which in turn reflect anatomical dimorphisms in the vocal apparatus between the two sexes. During male puberty, the testosterone-related growth of the laryngeal cartilages [4]–[6], and the associated lengthening and stiffening of the vocal folds [7], [8] cause men's F0 to drop by almost 50% compared to women's (men's F0: 120 Hz; women's: 200 Hz [8]), conferring men their characteristically lower-pitched voices. Moreover, the testosterone-induced differential body height, with men being on average 7% taller than women [9], coupled with the male-specific secondary descent of the larynx [10], result in men having longer vocal tracts and thus narrower ΔF (15–20% [11], [12]) than women, conferring a disproportionately more baritone quality to the male voice [10].

The voices of pre-pubertal children are also acoustically and perceptually different, and perceptual studies show that adults are able to correctly identify gender from the voice in children as young as four [3]. Several acoustic investigations have shown that, while children of both genders speak with similar F0s ([13]–[15]; but also see [16]) boys speak with lower formants and consequently narrower ΔF than girls [2], [3], [13], [14], [17], [18] despite the absence of overall differences in vocal tract length between the two sexes before puberty [10], [19]–[21]. This dimorphism has led to the suggestion that pre-pubertal sex differences in ΔF have a behavioural basis (for example boys may round their lips or lower their larynx when they speak to lengthen their vocal tracts – [2], [14]).

Taken together, these studies indicate that the between-sex dimorphism in the voice frequency characteristics (ΔF only in children and both ΔF and F0 in adults) is perceptually relevant to categorize the sex of speakers. Moreover, at least in adult voices, between-speaker variation in these parameters appears to also influence the perception of gender, a term which encompasses the biological and social attributes which a given society deems typical of either male (masculine attributes) or female (feminine attributes) sex [22]. For example, listeners consistently rate adult voices with naturally or artificially lower F0, lower ΔF, or both, as belonging to more masculine individuals than their raised versions [23], [24]. While variation in F0 and ΔF, which are both sexually dimorphic in adult voices, has been shown to influence listeners' attributions of adults' sex and gender characteristics, to our knowledge the effect of naturalistic variation in ΔF on sex and gender attributions has not been investigated in children's voices, despite the fact that this trait is sexually dimorphic.

Here we investigate whether small increments of ΔF in children's voices affect sex (male, female), as well as gender (masculine, feminine) attributions by adult listeners. In the first experiment (sex identification) we resynthesize ΔF along gender continua within the observed natural variation of this parameter and ask listeners to identify the sex of the speakers. We expect the identification function to be characterized by a gradual change from the male to the female category. In the second experiment (gender rating), we ask listeners to rate each voice stimulus on a scale that combines sex and gender information (from “masculine boy” to “feminine girl”). We expect that small, consecutive increments in ΔF will elicit a gradual increase in listeners' ratings from "masculine boy" to "feminine girl".

## Materials and Methods

### Ethics statement

Written consent from children's guardians as well as verbal consent from children were obtained prior to the recording of the voice stimuli. All adult subjects taking part in the psychoacoustic experiments gave written informed consent. Both procedures (voice recording and psychoacoustic experiments) were reviewed and approved by the Ethics Committee of the University of Sussex (authorization codes: DRVC0709 and DRVC0711).

### Subjects

252 second-year Psychology students (74 males, 178 females) from Sussex University took part in the psychoacoustic experiments (as part of their practical coursework in a Cognitive Psychology level two module). All subjects were fluent English speakers.

### Stimuli

Speech utterances were recorded using a *Shure SM94* microphone and a *Tascam DR07mkII* handheld recorder at a primary school in Sussex, as part of a previous study of gender expression in children's speech. During these recordings, two girls and two boys aged eight were asked to read out seven short words (“bed”, boot”, ”book”, “box”, “duck”, “hat”, “pig”). The recorded single-syllable words were individually standardized to 65 dB and concatenated prior to acoustic analysis and resynthesis.

### Acoustic analyses

We extracted F0 and formant frequencies using PRAAT v.5.1.19 freeware [25]. F0 was extracted using the command ‘to Pitch’, with analysis parameters set to: time-step 0.01 s; pitch floor, 60 Hz; pitch ceiling, 500 Hz. The frequency values of the first three formants (F_1_, F_2_, F_3_) were extracted using linear predictive coding (LPC) via the ‘LPC: To Formants (Burg)’ command, with analysis parameters set to: maximum number of formants, 5; maximum formant frequencies, 6000–6600 Hz; window of analysis, 0.025 s. Formant spacing ((1) ΔF = F_i+1_ - F_i_) was derived from F_1_–F_3_ values, by modelling the vocal tract as a uniform tube closed at the glottis and open at the mouth [26], [27]. Under such model, F_i_ are expressed as:
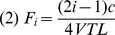



Where *i* is the formant number, *c* is the speed of sound in a mammal vocal tract (35,000 cm/s), VTL is the vocal tract length (in cm) and F_i_ is the frequency (in Hz) of *i*th formant. From (1) and (2), it follows that ΔF = F_i+1_ - F_i_ = *c*/2VTL (3). By replacing *c*/2VTL with ΔF in equation (2), ΔF can be derived as the slope of a regression model with the observed *F*_i_ values (y-axis) plotted against the expected formant positions:
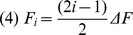
and the apparent vocal tract length (aVTL), as its inverse acoustic correlate measured in cm (aVTL = *c*/2ΔF). Therefore the longer the vocal tract, the lower the formant frequencies, and the narrower their overall frequency spacing. All extracted and derived acoustic values are reported in Table 1.

**Table 1:** Acoustic variables (F0, F_i_, ΔF in Hz) and apparent Vocal Tract Length (aVTL in cm) characterising the 4 exemplars (measured on concatenated strings of CVC words).

Exemplars	F0	F_1_	F_2_	F_3_	ΔF	aVTL
Girl 1	237	921	2125	3381	1383	12.7
Girl 2	304	859	2099	3370	1372	12.8
Boy 1	237	786	1933	3175	1283	13.6
Boy 2	262	768	2015	3194	1302	13.4

Average ΔF was 1377 Hz (aVTL 12.7 cm) for the two girl exemplars and 1293 Hz (aVTL 13.5 cm) for the two boy exemplars.

### Re-synthesis

**Figure 1:**
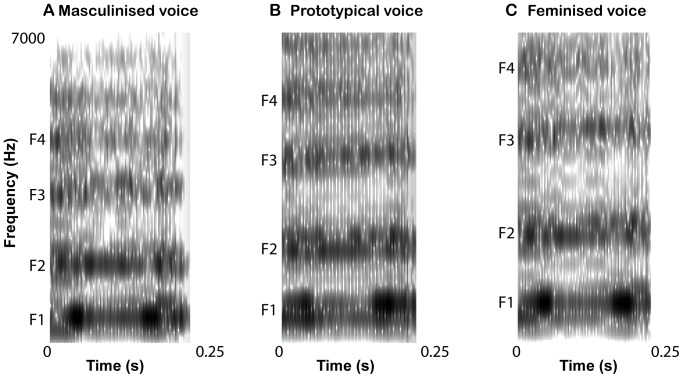
Spectrograms of vowel “” (from “book”) created from girl exemplar 1. Spectrogram settings: window length = .025 s, maximum number of formants, 5; maximum formant frequencies, 6000–6600 Hz. The formants (labeled F1–F4) are shifted down by 18% (A) and up by 12% (C) in comparison to the original signal (B), while all other acoustic parameters, including fundamental frequency, remain unchanged.

Following acoustic analysis, the stimuli were resynthesized using the "change gender" command in PRAAT. This command uses PSOLA, a resynthesis algorithm that allows the independent manipulation of formant frequency spacing (ΔF), mean fundamental frequency (F0), F0 variation and signal duration while keeping the values of all the other acoustic parameters (amplitude, noisiness etc.) unchanged. The mean fundamental frequencies were all standardised to 260 Hz (the average F0 measured in our sample). In order to remove possible intonation cues to gender, F0 variation was flattened by adjusting F0 values to the mean F0 (thus making the voice monotonous). Formant values were scaled up or down in increments of 2%, mimicking equivalent variations of ΔF (and thus aVTL) in speakers' voices. An increase of 2% of formant frequencies (achieved in the 102% stimuli) equates to a 2% increase in ΔF (corresponding to a 2% shortening of the vocal tract), and is expected to feminise the voice. As formant frequencies in our sample were on average 6% lower in the boy exemplars than in the girl exemplars, just below the gender difference reported in the literature for children of similar age (9–10% - [3], [18]) male voices were rescaled from 88% to 118%, while female voices were rescaled from 82% to 112%. The resulting continua were therefore not identical, but largely overlapping: the boys' continuum ranged from 1526 Hz to 1138 Hz (aVTLs from 11.5 cm to 15.5 cm), while the girls' continuum ranged from 1542 Hz to 1129 Hz (aVTLs from 11.4 cm to 15.5 cm). Supplementary online material includes audio files of example stimuli for one girl (Audio S1) and boy (Audio S2) exemplar. The resulting continua are within the range of ΔF variation observed in pre-pubertal children, as derived from published F1–F3 values [14], with aVTLs ranging from 11.4 cm to 15.9 cm for 5–12 year old children. They are also consistent with anatomical variation reported in [10], where VTLs for boys and girls, measured during quiet respiration, varied from 9.7 cm at age 5 to 14.0 cm at age 12. In summary, we generated 64 audio stimuli consisting of 16 re-synthesised variants of the single-syllable word lists by the two boys and the two girls. Figure 1 shows spectrograms of the vowel “υ” spoken by one of the exemplars, in which the formants (dark bands of energy in the spectrogram) are shifted compared to the original signal, while signal duration, F0 and F0 variation remain unchanged.

### Procedure

Participants completed the identification experiment first. Stimuli were presented using a PRAAT Multiple Forced Choice (MFC) experiment script and for each stimulus participants were asked to decide if the speaker was male or female (the instruction was: “Please identify the sex of the speaker”) by clicking the respective button on the screen (labelled “male” or “female”). A total of different 64 stimuli (16 variants from four exemplars) were presented once in a pseudo-random order. Participants were given an opportunity to pause after each series of 32 presentations. This experiment lasted approximately 10 minutes. In the second experiment, participants were asked to rate the same 64 voice stimuli from the sex identification task (also presented in a pseudo-random order using a MFC experiment script). The instruction was: “Rate the voice of the speaker on a scale of 1 to 7” and buttons were labelled as 1 = masculine boy, 2 = boy, 3 = feminine boy, 4 = neutral, 5 = masculine girl, 6 = girl, 7 = feminine girl.

### Statistical analyses

Because different sets of resynthesis variants (different formant scaling factors) were used for male and female exemplars, data are analysed and reported separately by exemplar's sex.

In order to test the effect of stimuli variant and listener sex on sex identification, we ran Generalised Linear Mixed Models (GLMM) with stimuli variant (scale), listener sex (nominal) and their interaction as fixed factors, exemplar id and subject id as random factors, and sex identification score (0 = male, 1 = female) as a binomial target variable. In order to test the effect of stimuli variant and listener sex on gender ratings we ran Linear Mixed Models (LMM) with stimuli variant (scale), listener sex (nominal) and their interactions as fixed factors, exemplar id and subject id as random factors, and gender rating as a scale outcome variable (from 1 = masculine boy to 7 = feminine girl).

Simple logistic regressions (one for boy exemplars and one for girl exemplars) were then used to illustrate the relationship between formant frequency spacing and identified sex with average score (over all participants) as the dependent variable and stimuli variant as the independent variable. Logistic models provide estimates for the slope of the category (here ‘male’ to ‘female’) transition (b1 coefficient, ranging between 0 and 1, with lower values reflecting steeper transitions) [28]–[30] and for the perceived category boundary (where 50% of stimuli are categorised a male, and 50% as female). The category boundary was computed using the formula -Ln(b0)/Ln(b1) where b0 is the constant of the logistic curve and b1 is the coefficient related to the slope 30,31. Simple linear regressions with stimuli variant as the predictor variable and average gender ratings (over all the participants) as the outcome variable were used to illustrate the relationship between formant frequency spacing variant and perceived gender. All the statistical analyses were performed using SPSS v.20.0.

## Results

### Sex identification experiment

**Figure 2:**
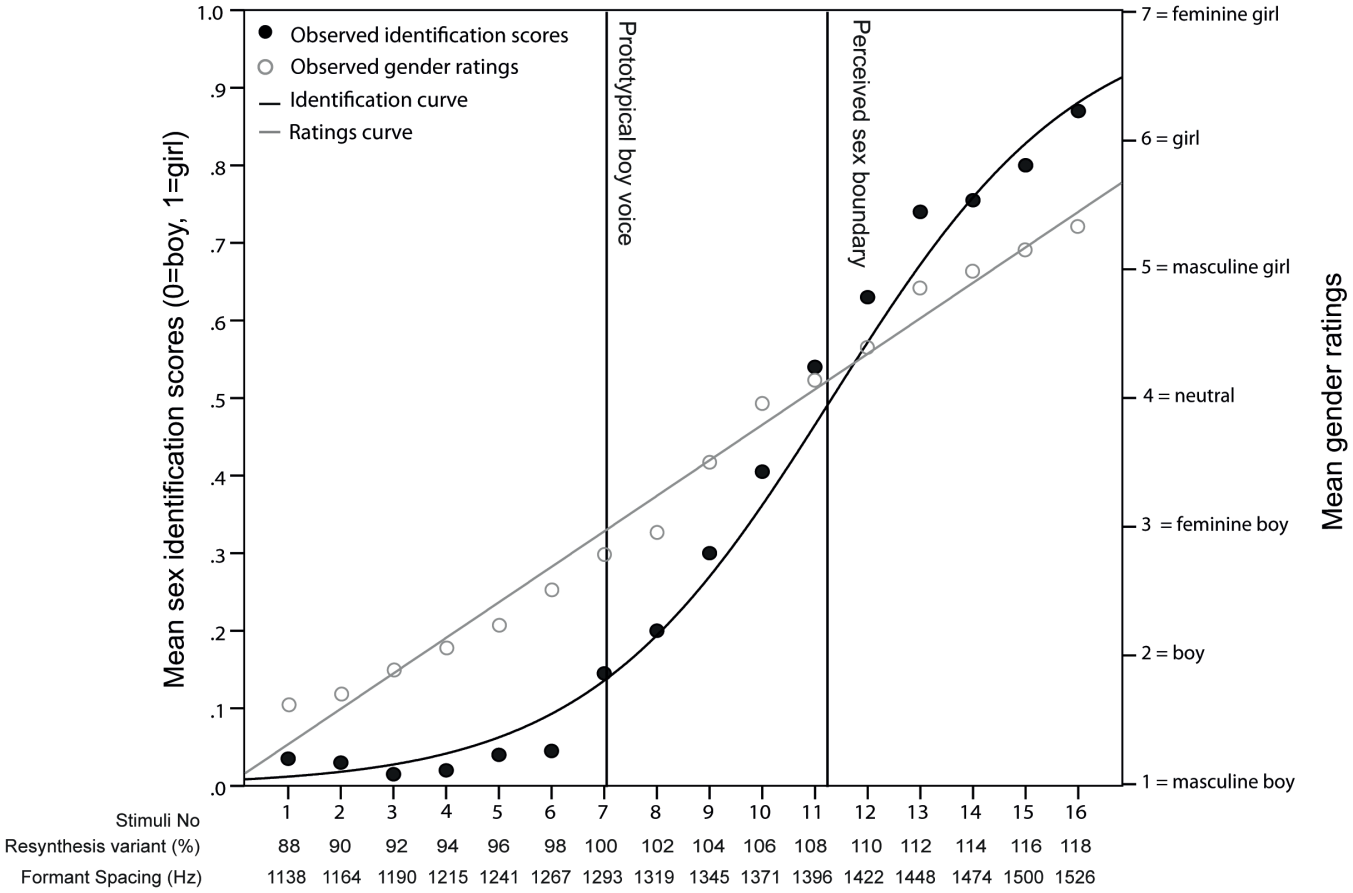
Identification and rating scores of boys' voices along the gender continua. Scores were averaged across listeners on voice stimuli (numbered 1–16 on the x-axis) for the boys' exemplars. The mean identification scores are plotted from 0 = male to 1 = female (left y-axis) and fitted with the logistic curve (black line). The vertical lines illustrate the location of the estimated sex boundary (where 50% of the listeners rate the stimuli as female) and the location of the prototypical boy voice stimulus (100%). The percentage of stimuli identified as female follows an S-shaped pattern along the continuum of resynthesis variants. The sex identification curve is characterised by a lower plateau for stimuli 1 to 6 (ΔFs of 1138–1267 Hz), where less than 10% of the stimuli are identified as female, indicating that stimuli variant with the lowest ΔF are mostly identified as male. The percentage of stimuli identified as female then increases gradually and linearly, and while no upper plateau is reached, average scores for stimuli 14 to 16 (ΔFs of 1474–1526 Hz) varied from 76% to 85%, indicating that boys' voices with the highest ΔF are mostly classified as female. Average gender rating scores are plotted from 1 = masculine boy (or girl) to 7 = feminine boy (or girl) (right y-axis) and fitted with a linear function (straight grey line). Mean gender ratings of male voices ranged from 1.78 (SE = .07) for the lowest ΔF variants to 5.36 (SE = .08) for the highest ΔF variants.

The results of the GLMM on sex identification scores of boy exemplars revealed a significant main effect of stimuli variant, F_1,8.060_ = 2,696.66, *p*<.001, while no significant main effects of listener's sex, F_1,8.060_ = 2.50, *p = *.114, and of its interaction with stimuli variant, F_1,8.060_ = 3.47, *p = *.063, were found. A logistic regression (Fig. 2 – black line) provided a strong statistical fit for the observed relationship between stimuli variant and average sex identification scores, R^2^ = .95, F_1,14_ = 240.43, *p*<.001. The relatively shallow transition (b1 = .65) from one response category to the other indicates that the percentage of stimuli identified as female increases progressively as ΔF increases. Using this model, the estimated ‘‘male-female” boundary fell between stimulus 11 and 12 (−Ln(127.43)/(.65) = 11.25, where b0 = 127.43 and b1 = .65, corresponding to 108%–110% variants or ΔF∼1400 Hz).

**Figure 3:**
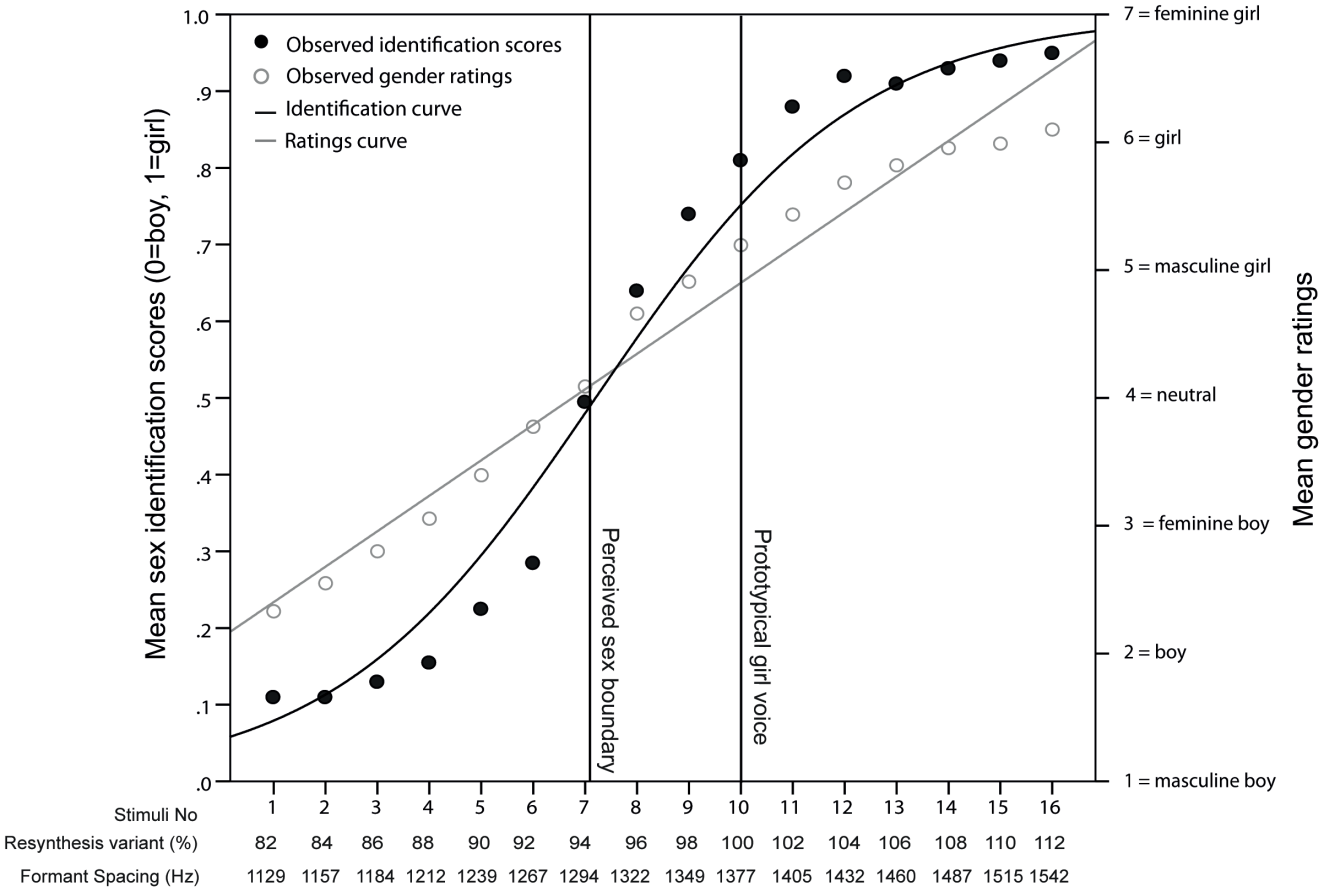
Identification and rating scores of girls' voices along the gender continua. Scores were averaged across listeners on voice stimuli (numbered 1–16 on the x-axis) for the girls' exemplars. The mean identification scores are plotted from 0 = male to 1 = female (left y-axis) and fitted with the logistic curve (black line). The vertical lines illustrate the location of the estimated sex boundary (where 50% of the listeners rate the stimuli as female) and the location of the prototypical boy voice stimulus (100%). The percentage of stimuli identified as female also follows an S-shaped pattern along the continuum of resynthesis variants. The sex identification curve is characterised by a lower plateau for stimuli 1 to 3 (ΔFs of 1129–1184 Hz), where between 10% and 15% of the stimuli are identified as female, indicating that stimuli variant with the lowest ΔF are mostly identified as male. The percentage of stimuli identified as female then increases gradually and linearly until it reaches an upper plateau from stimuli 12 to 16 (ΔFs of 1432–1542 Hz), with average scores varying from 92% to 95% and indicating that girl voices with the highest ΔF are mostly classified as female. Average gender rating scores are plotted from 1 = masculine boy (or girl) to 7 = feminine boy (or girl) (right y-axis) and fitted with a linear function (straight grey line). Mean gender ratings of female voices ranged from 2.33 (SE = .02) for the lowest ΔF variants to 6.10 (SE = .06) for the highest ΔF variants.

The results of the GLMM on sex identification scores of girl exemplars revealed a significant main effect of stimuli variant, F_1,8.060_ = 1,869.28, *p*<.001, while no significant main effects of listener's sex, F_1,8.060_ = 1.99, *p = *.158, and of its interaction with stimuli variant, F_1,8.060_ = 2.04, *p* = .153, were found. A logistic regression (Fig. 3 – black line) provided a strong statistical fit for the observed relationship between stimuli variant and average identification scores, R^2^ = .97, F_1,14_ = 382.14, *p*<.001. The relatively shallow transition (b1 = .67) from one response category to the other indicates that the percentage of stimuli identified as female increases progressively as ΔF increases. Using this model, the estimated ‘‘male-female” boundary fell between stimulus 7 and 8 (−Ln(17.37)/Ln(.67) = 7.13, where b0 = 17.37 and b1 = .67, corresponding to 94%–96% variants or ΔF∼1300 Hz).

### Gender rating experiment

The results of the LMM on gender ratings of boy exemplars revealed a significant main effect of stimuli variant, F_15,7781_ = 692.41, *p*<.001. No significant main effect of listener's sex, F_1,250_ = 2.24, *p = *.136, and of its interaction with stimuli variant, F_1,7781_ = 1.136, *p = *.317, were found. The results of the LMM on gender ratings of girl exemplars revealed a significant main effect of stimuli variant, F_15,7781_ = 626.87, *p*<.001. No significant main effect of listener's sex, F_1,250_ = .196, *p = *.658, and of its interaction with stimuli variant, F_1,7781_ = .714, *p = *.773, were found. Simple linear regressions (Figures 2 and 3 – grey straight lines) provided strong statistical fits for the observed correlation between variant number and average gender rating scores, showing that scores increased (from masculine boy to feminine girl) as formant frequency spacing increased (male exemplars: R^2^ = .99, F_1, 14_ = 893.04, *p*<.001, female exemplars: R^2^ = .97, F_1, 14_ = 459.94, *p*<.001).

## Discussion

The results of the sex identification and gender rating experiments show that ΔF is an important cue for the perception of sex and gender in the pre-pubertal human voice, in line with the previously reported acoustic dimorphism of this parameter in pre-pubertal speakers [8], [14], [17], [32]. More specifically, the absence of a sharp boundary between the sex categories in the identification experiment, in which listeners were asked to identify the child speaker as male or female, suggests that small, sex-related acoustic variation in ΔF proportionally affects the probability of voices to be perceived as either male or female by raters. Additionally, the gradual slope in voice ratings from “masculine boy” to “feminine girl” in the second experiment shows that small linear increments in ΔF also proportionally affect listeners' attributions of speakers' gender (from masculinity to femininity). Similar results have been reported in studies of gender perception in adult voices. A study using a combination of identification and discrimination paradigms [29] found that variations along a male-female continuum of F0 and ΔF, the main cues to sex in adult voices, were not remapped by listeners into separate psychological (male or female) categories, indicating that the perception of voice sex was not categorical. Moreover, psychoacoustic studies have shown that both men's and women's voices with naturally low, or artificially lowered, F0 and ΔF (or both), are rated as more masculine [23], [24], [33].

In the present study, while the resynthesis continua used for boy and girl exemplars were largely overlapping (boys: 1138–1526 Hz; girls: 1129–1542 Hz) and both comprised within the range of ΔF values achievable by both genders before puberty [10], [14], the effect of the rescaling of ΔF differed between boy and girl voice exemplars, suggesting that the resynthesis of this parameter was not sufficient to produce a voice systematically perceived as belonging to the opposite sex, despite the standardisation of F0 and its variation. In the sex identification experiment, the perceived sex boundary between male and female identification estimated by the logistic model is ∼100 Hz higher in boy voice exemplars than in girl voice exemplars (Figure 2 – vertical lines), revealing that a greater upward shift in ΔF was required for resynthesized stimuli from the voices of the two boy exemplars to be perceived as female. The identification curve (Figure 2 – black line) for the male exemplars is also shifted downwards relative to that of the female exemplars (Figure 3 – black line), with a wider plateau at the lower (male) end of the continuum, and no plateau at the upper (female) end of the continuum. Further, the boys' rating function (Figure 2 – grey straight line) from the gender rating experiment is shifted downwards compared to girls', revealing that stimuli from boy exemplars were perceived as more masculine than those from girl exemplars. One possible explanation for the observed perceptual differences is that listeners were affected by acoustic factors other than those manipulated (ΔF) or factored out (F0 and its variation) in the present experiments. For example, Klatt & Klatt [34] report that women are perceived to have more breathy voices than men, corresponding to increased F_1_ bandwidths and decreased F_1_ amplitude, while breathy voices are judged as more feminine than less-breathy voices [35], suggesting that, at least in adults, breathiness may be a contributing factor to the perception of sex and gender. The potential role of parameters such as F0, F0 variation and breathiness [8], [34], which are sexually dimorphic in adults, but not in pre-pubertal children [13]–[15], in the attribution of sex and gender to children's voices, is an important area for future research.

Independently from other hypothetical voice cues to sex and gender attributions of pre-pubertal children's voices, this study clearly identifies a substantial effect of ΔF variation on adults' ratings of gender in pre-pubertal speakers, with lower ΔF being consistently rated as belonging to more masculine children. ΔF variation has also been shown to affect judgements of body size and age in adult speakers, with listeners rating lower ΔF as belonging to older and larger individuals [36]–[39]. These perceptual differences in turn appear to relate to actual differences in age and size of speakers [39]–[41]. By extending the present paradigm to include age and body size ratings, future studies could investigate the perceptual linking of age-related size and gender dimensions, for example whether children that are perceived to be more masculine are also perceived to be older and bigger than their more feminine counterparts. Moreover, the use of natural (rather than re-synthesised) stimuli from children of different ages, body sizes and masculinities (i.e. as assessed by children's personal attributes questionnaires [42]), and of raters of different ages, would help clarifying the extent to which ΔF reliably cues for these dimensions throughout the lifespan.

Our observations that baseline ΔF variation within the natural range of children's voices affects listeners' sex and gender attributions (despite the absence of a clear anatomical basis for such variation) lends further support to the hypothesis that sex and gender expression in pre-pubertal children's voices have a strong behavioural, acquired dimension (with children learning to adjust their VTL in order to sound more or less feminine/masculine). Future studies using i.e. structural cine 3D structural MRI are now needed to further test this hypothesis.

Furthermore, it has been shown that children can also spontaneously modify ΔF (and F0) when asked to sound more or less like a boy or girl (Cartei, Cowles, Banerjee and Reby, unpublished data), suggesting that children can also control the gender-related characteristics of their voices. The extent to which this ability affects the expression of gender in everyday speech, in line with varying gendered roles (i.e. to affiliate with same-sex peers) and contexts (i.e. when speaking to a male or female), and its perceptual relevance in gendered attributions remains to be investigated.

## Supporting Information

Audio S1 **This audio file contains three variants derived from one of the two girl exemplar voices (exemplar 2), in which formant spacing was resynthesized from low (longer vocal tract – more masculine sounding voice) to high (shorter vocal tract – more feminine sounding voice) values (ΔFs: 88%,102%,110%).**
(WAV)

Audio S2 **This audio file contains three variants derived from one of the two boy exemplar voices (exemplar 4), in which formant spacing was resynthesized from low (longer vocal tract – more masculine sounding voice) to high (shorter vocal tract – more feminine sounding voice) values (ΔFs: 94%, 104%, 112%).**
(WAV)

## References

[1] Hillenbrand JM, Clark MJ (2009) The role of f0 and formant frequencies in distinguishing the voices of men and women. Attention, Perception, & Psychophysics 71: 1150–1166. 10.3758/APP.71.5.1150 19525544

[2] Sachs J, Lieberman P, Erickson D (1973) Anatomical and cultural determinants of male and female speech. In: Shuy RW, Fasold RW, editors. Language attitudes: Current trends and prospects. Washington,, DV: Georgetown University Press. pp. 74–84.

[3] Perry TL, Ohde RN, Ashmead DH (2001) The acoustic bases for gender identification from children's voices. The Journal of the Acoustical Society of America 109: 2988. 11425141 10.1121/1.1370525

[4] Hollien H, Green R, Massey K (1994) Longitudinal research on adolescent voice change in males. The Journal of the Acoustical Society of America 96: 2646. 7983270 10.1121/1.411275

[5] Dabbs Jr JM, Mallinger A (1999) High testosterone levels predict low voice pitchamong men. Personality and individual differences 27: 801–804.

[6] Rendall D, Kollias S, Ney C, Lloyd P (2005) Pitch (F) and formant profiles of human vowels and vowel-like baboon grunts: The role of vocalizer body size and voice-acoustic allometry. The Journal of the Acoustical Society of America 117: 944. 15759713 10.1121/1.1848011

[7] Hirano M, Kurita S, Nakashima T (1981) The structure of the vocal folds. In: Stevens K, Hirano M, editors. Vocal Fold Physiology. Tokyo: University of Tokyo Press. pp.33–41.

[8] Titze IR (2000) Principles of voice production. Iowa City, IA: National Center for Voice and Speech.

[9] Gaulin SJC, Boster JS (1985) Crosscultural differences in sexual dimorphism: Is there any variance to be explained? Ethology and Sociobiology 6: 219–225.

[10] Fitch WT, Giedd J (1999) Morphology and development of the human vocal tract: A study using magnetic resonance imaging. The Journal of the Acoustical Society of America 106. 10.1121/1.427148 10489707

[11] Fant G (1966) A note on vocal tract size factors and non-uniform F-pattern scalings. Speech Transmission Laboratory Quarterly Progress and Status Report 1: 22–30.

[12] Goldstein UG (1980) An articulatory model for the vocal tracts of growing children. Available: http://mit.dspace.org/bitstream/handle/1721.1/22386/Goldstein_Ursula_ScD_1980.pdf Accessed 2012 Jun 20.

[13] Busby PA, Plant GL (1995) Formant frequency values of vowels produced by preadolescent boys and girls. The Journal of the Acoustical Society of America 97: 2603–2606. 7714275 10.1121/1.412975

[14] Lee S, Potamianos A, Narayanan S (1999) Acoustics of children's speech: Developmental changes of temporal and spectral parameters. The Journal of the Acoustical Society of America 105: 1455. 10089598 10.1121/1.426686

[15] Sussman JE, Sapienza C (1994) Articulatory, developmental, and gender effects on measures of fundamental frequency and jitter. Journal of Voice 8: 145–156. 8061770 10.1016/s0892-1997(05)80306-6

[16] Tussey R, Canonaco J, Lynch C, Oss EV (2011) Out of the Mouths of Babes: Acoustic Variation in Child Speakers. 10th Annual Celebration for Undergraduate Research and Creative Performance (2011). Available: http://digitalcommons.hope.edu/curcp_10/129.

[17] Whiteside SP, Hodgson C (2000) Some acoustic characteristics in the voices of 6- to 10-year-old children and adults: a comparative sex and developmental perspective. Logopedics Phoniatrics Vocology 25: 122–132. 10.1080/14015430050175851 11086804

[18] Bennett S (1981) Vowel formant frequency characteristics of preadolescent males and females, The Journal of the Acoustical Society of America. 69: 321–238. 10.1121/1.385343 7217521

[19] Vorperian HK, Kent RD (2007) Vowel acoustic space development in children: a synthesis of acoustic and anatomic data. Journal of Speech, Language and Hearing Research 50: 1510. 10.1044/1092-4388(2007/104) PMC2597712 18055771

[20] Vorperian HK, Wang S, Chung MK, Schimek EM, Durtschi RB, et al (2009) Anatomic development of the oral and pharyngeal portions of the vocal tract: An imaging study. The Journal of the Acoustical Society of America 125: 1666–1678. 19275324 10.1121/1.3075589 PMC2669667

[21] Vorperian HK, Wang S, Schimek EM, Durtschi RB, Kent RD, et al (2011) Developmental sexual dimorphism of the oral and pharyngeal portions of the vocal tract: An imaging study. Journal of Speech, Language and Hearing Research 54: 995. 10.1044/1092-4388(2010/10-0097) PMC3135757 21106698

[22] Jackson S (1998) Theorising gender and sexuality. Contemporary feminist theories: 131–146.

[23] Pisanski K, Rendall D (2011) The prioritization of voice fundamental frequency or formants in listeners' assessments of speaker size, masculinity, and attractiveness. The Journal of the Acoustical Society of America 129: 2201. 21476675 10.1121/1.3552866

[24] Pisanski K, Mishra S, Rendall D (2012) The evolved psychology of voice: evaluating interrelationships in listeners' assessments of the size, masculinity, and attractiveness of unseen speakers. Evolution and Human Behavior.

[25] Boersma P (2001) Praat, A System for doing Phonetics by Computer. Glot International 5: 341–345.

[26] Cartei V, Cowles HW, Reby D (2012) Spontaneous Voice Gender Imitation Abilities in Adult Speakers. PloS one 7: e31353. 22363628 10.1371/journal.pone.0031353 PMC3281965

[27] Reby D, McComb K (2003) Anatomical constraints generate honesty: acoustic cues to age and weight in the roars of red deer stags. Animal behaviour 65: 519–530.

[28] Smits R, Sereno J, Jongman A (2006) Categorization of sounds. Journal of Experimental Psychology Human Perception and Performance 32: 733. 16822135 10.1037/0096-1523.32.3.733

[29] Mullennix JW, Johnson KA, Topcu-Durgun M, Farnsworth LM (1995) The perceptual representation of voice gender. The Journal of the Acoustical Society of America 98: 3080. 8550934 10.1121/1.413832

[30] Keating P (2004) Analysis of identification data. Available: http://www.linguistics.ucl.edu/faciliti/facilities/statistics.ident.htm. Accessed 2010 Jan 31.

[31] Aliaga-García C, Mora JC (2009) Assessing the effects of phonetic training on L2 sound perception and production. Recent research in second language phonetics/phonology: Perception and production. In: Watkins MA, Rauber AS, Baptista BO, editors. Newcastle upon Tyne: Cambridge Scholars Publishing. pp. 2–31.

[32] Whiteside SP (2001) Sex-specific fundamental and formant frequency patterns in a cross-sectional study. The Journal of the Acoustical Society of America 110: 464. 11508971 10.1121/1.1379087

[33] Munson B (2007) The Acoustic Correlates of Perceived Masculinity, Perceived Femininity, and Perceived Sexual Orientation. Language and Speech 50: 125–142. 17518106 10.1177/00238309070500010601

[34] Klatt DH, Klatt LC (1990) Analysis, synthesis, and perception of voice quality variations among female and male talkers. the Journal of the Acoustical Society of America 87: 820. 2137837 10.1121/1.398894

[35] Van Borsel J, Janssens J, De Bodt M (2009) Breathiness as a feminine voice characteristic: a perceptual approach. Journal of Voice 23: 291–294. 18675534 10.1016/j.jvoice.2007.08.002

[36] Smith DR, Patterson RD, Turner R, Kawahara H, Irino T (2005) The processing and perception of size information in speech sounds. The Journal of the Acoustical Society of America 117: 305. 15704423 10.1121/1.1828637 PMC2346562

[37] Rendall D, Vokey JR, Nemeth C (2007) Lifting the curtain on the Wizard of Oz: biased voice-based impressions of speaker size. Journal of Experimental Psychology-Human Perception and Performance 33: 1208–1219. 17924818 10.1037/0096-1523.33.5.1208

[38] Simmons LW, Peters M, Rhodes G (2011) Low pitched voices are perceived as masculine and attractive but do they predict semen quality in men? PloS one 6: e29271. 22216228 10.1371/journal.pone.0029271 PMC3244455

[39] Collins SA, Missing C (2003) Vocal and visual attractiveness are related in women. Animal Behaviour 65: 997–1004.

[40] Bruckert L, Lienard J-S, Lacroix A, Kreutzer M, Leboucher G (2006) Women use voice parameters to assess men's characteristics. Proc Biol Sci 273: 83–89. 16519239 10.1098/rspb.2005.3265 PMC1560007

[41] Rendall D, Kollias S, Ney C, Lloyd P (2005) Pitch (F) and formant profiles of human vowels and vowel-like baboon grunts: The role of vocalizer body size and voice-acoustic allometry. The Journal of the Acoustical Society of America 117: 944. 15759713 10.1121/1.1848011

[42] Hall JA, Halberstadt AG (1980) Masculinity and femininity in children: Development of the Children's Personal Attributes Questionnaire. Developmental Psychology 16: 270.

